# Delayed Hemorrhage in Kidney Transplantation: A Life-threatening Condition

**Published:** 2018-02-01

**Authors:** S. Gooran, A. Javid, G. Pourmand

**Affiliations:** 1 Urology Research Center, Sina Hospital, Tehran University of Medical Sciences, Tehran, Iran; 2 Alzahra Hospital, Isfahan University of Medical Sciences, Isfahan, Iran

**Keywords:** Renal transplantation, Aneurysm, Aneurysm, false, Post-operative hemorrhage

## Abstract

One of the most catastrophic complications of kidney transplantation is non-traumatic delayed bleeding caused by arterial dissection and pseudoaneurysm, endangering the survival of the graft and the patient. Herein, we discuss the management of this condition in 3 cases.

The patients included 2 men, 30 and 47 years old, and a 33-year-old woman, who developed a massive hemorrhage in the second week after kidney transplant. All our patients were diabetic for more than 5 years. Massive hemorrhage occurred in the second week without any trauma or precipitating factor. A combination of antibiotic therapy, surgery and interventional procedures was required and all three transplanted kidneys inevitably had to be removed. Although there were trivial signs of infection, considerable pus and infectious and necrotic tissue were drained during graft nephrectomy. A high index of suspicion is necessary for the timely diagnosis of arterial dissection and aneurysm. Aggressive treatment with arterial drug-eluting stents and surgical drainage are necessary in order to prevent potentially fatal complications.

## INTRODUCTION

Delayed bleeding caused by dissection of arterial anastomosis is a life threatening event after kidney transplantation [1, 2]. Renal artery dissection and pseudoaneurysms in the absence of endovascular procedures have been reported after renal transplantation and may occur due to adjacent pelvic infections [3, 4]. Although this is a rare complication, its importance cannot be overemphasized considering catastrophic consequences including sudden death [5, 6]. Early recognition of this entity based on a high index of suspicion and subsequently employing the most appropriate treatment are vital for its successful management.

There are few small series and isolated case reports of this potentially life-threatening condition in medical literature and hence there are limited data about its etiology, indications of intervention, management options, and prognosis. Herein, we report three patients suffering from this life-threatening complication and present the therapeutic procedures and the outcomes of the patients.

Case 1 

**Figure 1:**
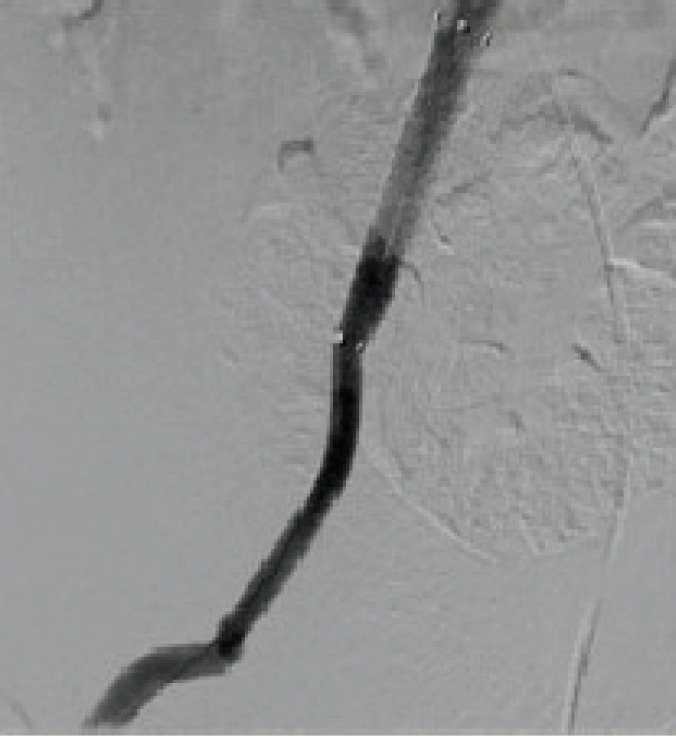
Drug-eluting stent graft in common and external illiac artery

A 30-year-old man with a 10-year history of end-stage renal disease due to diabetic nephropathy received a deceased renal transplant. There was a hemoglobin decline from 12 to 8.3 mg/dL. The ultrasound scan revealed a hematoma around the kidney on post-operative day 2, but the patient was hemodynamically stable and no active bleeding was reported. Two weeks after an uneventful transplantation and four days after hospital discharge, the patient returned to hospital with graft pain, tachycardia and low blood pressure. Color ultrasonography disclosed a massive bleeding of the grafted renal artery. The patient was hydrated and four units of blood were transfused; the patient was transferred to cath lab for angiography and placement of arterial stent graft (Fig 1). After placement of a drug-eluting stent graft in the external iliac artery sacrificing the renal transplant, the patient transferred to the operating room and the transplanted kidney was removed. The culture of necrotic tissue showed *Staphylococcus aureus* infection. Appropriate antibiotic treatment was started and the patient returned to a regular hemodialysis program.

Case 2

A 33-year-old woman with history of diabetes mellitus for 16 years and history of preeclampsia three years before, received a deceased renal transplant two years after commencing hemodialysis. Her primary renal disease was diabetic nephropathy. The procedure was uneventful and her serum creatinine level five days after surgery was 1.3 mg/dL. Suddenly, a massive bleeding started from the drain and the patient lost 1200 mL of blood. The patient, however, was hemodynamically stable and the bleeding stopped without any interventions. Ultrasonography revealed a massive hematoma around the transplanted kidney. There was no leakage or pseudoaneurism in angiography. The patient transferred to the operating room to evacuate the hematoma. Meanwhile, bleeding started from the trunk of the renal artery. We clamped the artery and repaired the laceration. One day after the repair, the patient developed acute anuria. A duplex scan was performed and showed renal artery thrombosis and a large hematoma around the kidney. Therefore, graft nephrectomy was performed. Before graft nephrectomy, renal thrombosis was confirmed by angiography in cath lab, but the interventionist refused to place a stent graft in common iliac artery. At exploration, a nonviable allograft surrounded by about 1 L of thick pus was removed and mixed bacterial flora of *S. aureus* and *S. epidermidis* grew in the culture. The patient died of abrupt hemorrhage four days later, while she was receiving broad-spectrum antibiotics.

Case 3

**Figure 2:**
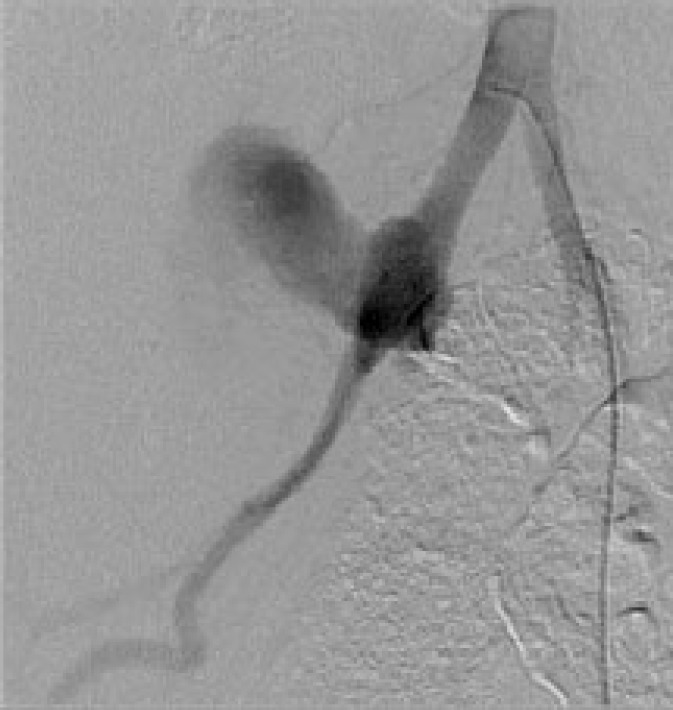
Arterial dissection in grafted renal artery

A 47-year-old man with a history of diabetes mellitus, hypertension, and 10 years of hemodialysis underwent kidney transplantation from a 42-year-old deceased donor. Five days after the surgery, the patient experienced bleeding from surgical site and hemoglobin decline. The patient was managed conservatively by complete bed rest, blood transfusion, and injection of tranexamic acid. One week later, massive hemorrhage was suddenly repeated. The patient transferred to cath lab for angiography (Fig 2) and arterial bypass was performed using stent-graft at the anastomosis. The massive bleeding was successfully stopped. The patient was transferred to the operating room for graft nephrectomy. The culture taken was positive for *Candida albicans*. Antibiotic and antifungal agents were administered in ICU. The patient discharged from the hospital and became a candidate for the next renal transplantation.

## DISCUSSION

These cases showed that non-traumatic hemorrhage after renal transplantation is a hazardous event that may occur repeatedly. Hemoglobin decline and hematoma around the transplanted kidney—which were present in all our cases before arterial dissection—were associated with a significant risk of infection. In our cases, we encountered pus and necrotic tissue before nephrectomy and the surgical field was obviously infected. We think the infection of the artery at or around the site of arterial anastomosis may lead to an anastomotic leak or local dissolution of the arterial wall and consequently, carry the potential of rupture with catastrophic consequences. We therefore believe that hematoma and pus should be drained early to prevent morbidity and mortality.

All our patients were diabetic for more than five years. The masking effect of diabetes and immunosuppression were clearly demonstrated in our cases. Apart from a raised white cell count, there was no sign of serious infection until the abrupt hemorrhage from the anastomotic patch.

The choice of treatment strategy is still a subject of debate, requiring further delineation. Some authors have recommended immediate graft nephrectomy as the treatment of choice of extra-renal arterial dissection after renal transplant [7]. On the other hand, re-suturing or re-anastomosis is contraindicated in a contaminated field.

Endovascular placement of drug-eluting stent grafts is a valuable therapeutic option in cases of renal artery dissection and pseudoaneurysms after renal transplantation. It is relatively safe and easy to perform, with good early and long-term results and allows graft nephrectomy to be performed in a safer manner. The placement of drug-eluting stent grafts overcame the risk of bleeding and allowed graft nephrectomy to be performed more easily and safely [8].

To prevent non-traumatic massive hemorrhage after renal transplantation due to arterial dissection or pseudoaneurism, it is necessary to notice post-operative hematomas and surgical field infections. We prescribe longer courses of perioperative antibiotics in susceptible patients with diabetes mellitus and perirenal hematoma. Massive perirenal hematomas should be evacuated as soon as possible to prevent superimposed infection. *S. aureus* was found in two and *C. albicans* in another patient. Prophylaxis with a broad-spectrum antibiotic active against staphylococcus given at induction of the anesthesia has been recommended to be of benefit in peripheral vascular surgery [9, 10]. We recommend this approach in diabetic patients too.

When nephrectomy is indicated, drug-eluting stent placement in common iliac and external iliac artery is recommended followed by graft nephrectomy. 

In conclusion, delayed bleeding after kidney transplantation is a catastrophic complication that can occur repeatedly and hence requires aggressive treatment. 

## CONFLICTS OF INTEREST:

None declared.
